# Active Commuting Behaviours from High School to University in Chile: A Retrospective Study

**DOI:** 10.3390/ijerph16010053

**Published:** 2018-12-26

**Authors:** Maribel Parra-Saldías, Jose Castro-Piñero, Antonio Castillo Paredes, Ximena Palma Leal, Ximena Díaz Martínez, Fernando Rodríguez-Rodríguez

**Affiliations:** 1 Grupo IRyS, School of Physical Education, Pontificia Universidad Católica de Valparaíso, Viña de Mar 2520000, Chile; fernando.rodriguez@pucv.cl; 2 Department of Physical Education, Faculty of Education Sciences, University of Cadiz, Puerto Real 11519, Spain; jose.castro@uca.es; 3 School of Pedagogy in Physical Education, Faculty of Education, University of the Americas, Santiago 8370035, Chile; acastillop85@gmail.com; 4 School of Doctorate and Research, Doctoral Program in Physical Activity and Sports, European University of Madrid, Madrid 28670, Spain; 5 Department of Physical Education, Sports and Recreation, Technical University Federico Santa María, Valparaíso 2390123, Chile; ximena.palmaleal@gmail.com; 6 Quality of Life Research Group, Department of Education Sciences, Universidad del Biobío, Chillán 3780000, Chile; xdiaz@ubiobio.cl

**Keywords:** active transport, high school, students, physical activity

## Abstract

*Objective*: To compare the differences in the modes and distance of the displacements in high school and university stage in the same sample. *Methods*: A total of 1288 volunteer university students (614 males and 674 females) participated, with an average age of 22.7 ± 5.8 years, belonging to four private and public universities in Chile where a validated self-report questionnaire was applied to the study, which included the modes, travel time, and distance at school and university. *Results*: The active commuting decreases from school to university when leaving home (males: 39.6% to 34.0%; *p* = 0.033 and females: 32.9% to 18.5%, *p* < 0.001), as well as when returning (males: 44.1% to 33.7%; *p* < 0.001 and females: 38.6% to 17.6%, *p* < 0.001). Conversely, non-active modes of transport increase, especially in females (go: 67.1% to 81.4%, return: 61.5% to 82.6%), affected by the increase in the use of public transportation in university. It was also defined that at both school and at university, the active commuting decreases the greater the distance travelled. *Conclusion*: The active modes of commuting decreased between high school and university and the non-active mode of commuting was the most frequent form of mobility to high school and university, observing that the active trips decreased when the distance from the home to high school or university increased. Public and private intervention policies and strategies are required to maintain or increase the modes of active commuting in the university stage for an active life in adulthood.

## 1. Introduction

From childhood to adolescence, the practice of physical activity (PA) suffers a decline [1,2,3], this tendency is much more marked in females [4], becoming a model that remains until adulthood, characterized by the progressive abandonment of this lifestyle over time [5,6]. The time between high school and university represents one of the great transitions of life [7], which requires the student multiple changes and adaptations in their daily routine; characterized by increased sedentary lifestyle and a marked decrease in physical activity [8,9,10].

Some authors agree that in the last phase of adolescence and in the first phase of adulthood, there is a pronounced decrease in PA levels [10,11]. A cross-sectional study of school and university students showed that 72% of children and 56.4% of adolescents met the PA recommendations of the World Health Organization (WHO), compared with 40% of compliance of university students [12]. Another study indicates that only 43% of university students have sufficient levels of PA, being different by gender, 55% males and 34% females [13], demonstrating a decrease in the levels of PA or even abandonment of exercise practice [5].

On the other hand, during the university stage, compulsory physical education class disappears and young people use their time in more sedentary activities (e.g. passive transportation, sitting to study, internet use, and sitting to hang out/talk with friends/family) [14]. An adequate urban infrastructure, good sidewalks, and bicycle paths in university environments, together with strategies of promotion, would be environmental factors to increase the practice of PA during leisure time. In this sense, active commuting (AC), walking or cycling, can contribute to the increase in total daily PA [15,16] and increase the PA level of the population [17,18].

The link between AC and the increase in PA associated with health benefits has been clear for some years, they have reported decreases in cardiovascular disease rates, [19] type 2 diabetes [20], and mortality associated with the use of bicycles [21]. It is also possible to mention other benefits as it contributes to the reduction of car congestion [22,23] and the reduction of atmospheric pollution [24,25].

Several studies have examined the commuting patterns of university students. The way of commuting can be influenced by the distance between the place of residence and the university, and is an important aspect when deciding the mode of commuting. University students who lived less than 2 km from the university generally walked, and when the distance from home to the university was 2 to 5 km, they preferred the use of bicycles [26,27,28], that is, studying or working less than 5 km from home was positively associated with active modes of transport [29]. Moreover, AC is more common than car conduction in the university, when the journey took less than 20 min [30]. Two prospective European studies, performed in small samples, found that AC decreased during the transition from high school to university [14,31]. It would be desirable to know if these findings also occur in developing countries. According to our knowledge, there is no scientific evidence on AC comparing active commuting behaviours between high school and university students in Chile.

Therefore, the purpose of the present study was to compare the differences in the modes and distance of the displacements in the high school and university stage in the same sample.

## 2. Materials and Methods

### 2.1. Study Design and Participants

This research is a retrospective and cross-sectional study, with a purposive sample for convenience, composed of 1288 volunteer university students (614 males and 674 females), with an average age of 22.7 ± 5.8 years, carried out in four different private and public universities, located in four Chilean urban cities (Valparaíso, Viña del Mar, Santiago, and Chillán), corresponding to the Universidad del Bío Bío (UBB), Universidad Técnica Federico Santa María (UTFSM), Pontificia Universidad Católica de Valparaíso (PUCV), and Universidad de Las Americas (UDLA), respectively. The students belonged to diverse faculties (art, engineering, sciences, and education) attending the first three years of university studies. The range of stay of the university students was from one to five years.

### 2.2. Instruments

The questionnaire applied was created at the University of Granada (Spain) (http://profith.ugr.es/paco), through the project "PACO: Pedaling and walking to school". This questionnaire was the result of a systematic review of 158 studies in the scientific literature about the evaluation of the modality of transfer using questionnaires [32]. This language used in this instrument has been adapted to be applied in the Chilean reality for university students. Additionally, a test-retest reliability test (two applications separated by seven days) was carried out, which obtained a Kappa coefficient (k) for AC modes of 0.946 (*p* = 0.277) and an intraclass correlation coefficient (ICC) for distance of 0.963 (IC = 0.946–0.975) and for time of 0.976 (IC = 0.966–0.984). The questionnaire included closed questions with multiple answers on sociodemographic variables; socioeconomic level, where the family affluence scale (FAS) was used [33]; and questions about the mode of travel [34]. The way of commuting was measured by asking participants how they usually travelled to and from the high school (last year) or university. The response options were as follows: walk, cycle, car, motorcycle, public bus, and metro/train. When participants used mixed mode transport (e.g. walk to bus), the mode of transport was assigned based on the longest portion of their trip. For commuting distance to and from home to high school or university, participants could choose any of the following options: 0 to 1.5, 1.51 to 4, 4.1 to 10, and ≥ 10.1 km for high school; and 0 to 2, 2.1 to 6, 6.1 to 15, and ≥ 15.1 km for university.

Informed consent was given to be read and signed by the volunteers who agreed to participate in the study. Later, the questionnaire was applied at the beginning of the classes of the respective subjects of the university students who participated in the study. 

### 2.3. Procedure 

Before the start the project, in order to comply with the legal ethical criteria, a letter was sent to the corresponding authorities of the different universities explaining the objectives of the study. Once the authorization was obtained by the authorities, the students were informed of the different courses and subjects. Volunteers who agreed to participate in the study were given informed consent for their reading and signature. The application of the questionnaire in paper format was answered at the beginning of the classes of the respective subjects where the study was accepted. The teacher who collaborated with the application of the survey informed the volunteers that the information given for it was confidential. All belonged to the first years of their respective careers. The study was applied in four Chilean universities (public and private) during the months of April and June of 2017. All this lasted approximately 15 to 30 min.

### 2.4. Ethical Aspects

The informed consent provided explained the characteristics of the questionnaire, the purpose of the study, and the confidentiality of the results. All the participants voluntarily agreed to the study, which was approved by the Ethics Committee of the corresponding university (Code: CCF02052017) and governed by the Helsinky 2004 declaration [35]. 

### 2.5. Statistical Analysis

Descriptive analyzes were performed as mean and standard deviation for socioeconomic aspects, university, and study area. The mode of commuting used by students from home to university, and vice versa, is presented as a number (percentage). Differences in the commuting mode between school and university students were analyzed by McNemar test, divided by gender. The mode of commuting was dichotomized as active (walking and cycling) or not active (car, motorcycle, public bus, and metro/train). A multinomial logistic regression analysis was performed to examine the association of distance groups with mode of commuting (i.e. active/non-active change). As there were no interactions of gender groups with the mode of commuting, all analyzes were performed jointly for males and females, adjusted by gender.

The statistical analyzes were conducted using the IBM SPSS Statistics (v. 21.0 for WINDOWS, Chicago, IL, USA.), and the level of significance was set to *p* < 0.05.

## 3. Results

Table 1 presents the general characteristics of the students who participated in the study, separated by gender, university, and area of study. The largest proportion of the sample was in an average socioeconomic level (52.8%), according to the FAS scale. The study areas of origin of the students were mainly engineering and health sciences (36.5% and 22.8%, respectively).

Table 2 shows AC and non-active mode of commuting of students from home to high school or university, separated by gender. The modes of AC showed a significant decrease from high school to university (32.9% vs. 18.5%; *p* < 0.001) in females. Walking, which was the main active mode in high school, decreased significantly from 32.6% to 17.5% (*p* < 0.001) in females. At the same time, there was an increase in non-active commutingnon-active commuting when comparing high school and university (67.1% vs. 81.4%; *p* < 0.001), especially influenced by the increase in public transport use: public bus (31.8% vs. 42.5%; *p* < 0.001) and metro/train (4.1% to 10.4%, *p* < 0.001).

In males, AC decreased significantly when going from high school to university (39.8% to 34.4%; *p* = 0.039). The use of private cars significantly decreased (23.9% vs. 12.1%; *p* < 0.001); however, the use of the public bus and metro/train increased (all *p* < 0.001) from high school to university.

Table 3 shows the differences in returning home from high school or university by gender. The decrease in active commuting can be observed in males from 44.1% to 33.7% (*p* < 0.001) and in females from 38.6% to 17.6% (*p* < 0.001). Walking prevailed as the main means of active commuting, decreasing in males from 41.0% to 31.3% (*p* < 0.001) and in females from 38.0% to 16.9% (*p* < 0.001). There was an increase in non-active commuting in both genders (males: 55.9% vs. 66.2%, *p* < 0.001; females: 61.5% to 82.6%, *p* < 0.001).

Table 4 shows the probability of being active or non-active, according to distance from high school and university to home and vice versa. The distance was greater when moving to university, establishing the first quartile (Q1) of distance from 0 to 2 km, unlike the transfer to high school, where Q1 is from 0 to 1.5 km. The odds ratio (OR) of being non-active in high school and university increased when the distance was higher (5.48 in Q2, 35.62 in Q3, and 205.04 in Q4 for high school; and 31.20 in Q2, 86.298 in Q3, and 207.40 in Q4 for university; all *p* < 0.001). This phenomenon also occurred back home, with the OR = 6.95 and 22.74 in Q2 (all *p* < 0.001) for high school and university, respectively; rising exponentially as the distance increased (37.44 in Q3 and 239.01 in Q4 for high school; and 132.76 in Q3 and 150.92 in Q4 for university; all *p* < 0.001).

There was no association between the years of stay and the mode of commuting or type of transport (data not shown).

## 4. Discussion

### 4.1. Differences in Commuting Mode between High School and University

AC in university students has not been sufficiently studied, even though the benefits of this mode of commuting on the health of people are well documented [36,37,38,39]. 

This study has presented the differences in the modes of commuting between high school and university, where AC decreased from high school to university (39.8% vs. 34.0% in males and 32.9% vs. 18.5% in females from home to high school/university; and 44.1% vs. 33.7% in males and 38.6 vs. 17.6% in females from high school/university to home). Despite this decrease, the results of high school AC were higher than those previously reported in Chilean schoolchildren, where only 10% of children and around 25% of adolescents go to school actively [40]. These results are consistent with previous prospective studies in males [14,31], but not in females, where we found a greater decrease in AC. Deforche et al. [14] did not find differences between genders, however, Garcia-Molina et al. [31] showed that AC levels only decreased in males and remained the same in females. To our knowledge, no study has analyzed the causes of these gender differences. Future studies are needed to investigate which individual, psychosocial, and environmental factors could determine gender differences in AC from high school to university.

It is known that walking reduces the risk of cardiovascular diseases and chronic non-contagious diseases, such as being overweight, obesity, hypertension, and diabetes, among others [41], obtaining a reduction of up to 11% of cardiovascular risk in males and females who move actively [42]. Commuting actively to the place of study for more than 15 minutes per day is a recommended behavior that is associated with a higher level of happiness and well-being in adolescence [43]. Moreover, AC helps to counteract the current low levels of PA [37] and can provide multiple physiological benefits to health [36,44]. Unfortunately, we found a low prevalence of AC walking in both groups (high school and university), which was almost 50% lower in university students.

On the other hand, the proportion of students who use bicycles as an active mode to and from high school or university was around 3% in males and did not reach 1% in females, which are low values compared with other studies that showed a prevalence of 10.6% in Spain [45] and 6% in Australian [46] university students, in both genders. Likewise, other less hilly cities in Chile reported a higher use of bicycles in adults, such as Rancagua and Los Angeles (4%), Curico (12%), Talca, and Chillán (8%) [47]. The link between the use of bicycles and health benefits has been shown in several studies, through systematic reports accounting for the reduction in cardiovascular diseases [19], type 2 diabetes [20,48], and mortality [21], thus the AC modes adopted by university students could contribute to a better quality of life. On this subject, the surroundings of the university have a great influence on the choice of the students’ commuting mode [39], also knowing that universities have the responsibility to create favorable environments to promote the health of their students and increase habits of active life [27,49,50]. 

According to non-active commuting, a high prevalence was observed in both high school and university, increasing from high school and university (60.3% to 65.8% in males and 67.1% to 81.4% in females, from home to high school/university; 55.9% to 62.2% in males and 61.5% to 82.6% in females, from high school/university to home). This affects the PA levels of the university students, given that the opportunity to perform AC is low. It is important to consider that the transition to adulthood is related to the decrease in active commuting mode and increase in the use of public transport [31], which may reduce the total physical activity levels. A North American study conducted at Kansas State University found a prevalence of non-active commuting of 34.7% [30], while a study conducted on 518 students from two universities in Spain showed a prevalence of 65.1% of non-active commuting [26]. This study reported that the metro/train was the main non-active commuting mode. In the same sense, an Australian study obtained 71% non-active mode commuting (32% train, 22% car, 17% bus) [46]. However, in the present study, the public transport used the most was the public bus (≈40% in high school and ≈50% in university) as opposed to the metro/train (≈3% in high school and ≈10% in university). These percentages did not change by high school or university, except in Chillan where there is no metro/train. These use of public buses increased significantly from high school to university, which could contribute to improved AC, as this type of commuting mode implies walking to reach the point or stop of public transport [51,52]. A study in a Spanish university estimated that those using public transportation to go to university could incorporate a total of 96 min/week of physical activity [26], which can make a large difference in annual energy expenditure.

### 4.2. Travel Distance between School and University

Distance is one of the main obstacles for AC. Previous research has shown that people who live closer to work are more likely to walk [53]. It has also been shown that for every additional 1 km between home and the place of work, the probability of walking and riding a bicycle is reduced by 3.9 times and 1.3 times, respectively [54,55].

This indicator of the probability of using an active or a non-active mode from home to high school or university negatively influences the choice of mode of transport. Distance is one of the main barriers to AC [40], making it difficult to use a bicycle or to take walks in urban or rural environments [56], or in places where public transport is scarce or of poor quality. Walking is a modifiable behavior that can become the predominant form of transport for travel at a certain distance. In a Spanish study, a threshold was determined for active commuting to school and back home of 875 m in children and 1.350 km in adolescents [57], while in adults, the threshold was less than 1.6 km [58], coinciding with our findings.

The results of this study show that as the distance of commuting increased, the probabilities of choosing a non-active commuting mode increased in both high school and university, with the distance to university being greater, which resulted in less AC compared with high school. A study carried out by the Autonomous University of Barcelona justified the use of non-active commuting modes, owing to the large distances from the university campuses and because the infrastructure was available only for motorized transport [59].

The use of bicycles in the present study was higher in males (≈3.2% in high school and ≈2.0 in university) than in females (≈0.5% in high school and ≈0.8% in university). This low prevalence could be influenced by several factors. For instance, a Latin American study indicated a lack of a favorable built environment, as there was only one bicycle path that represented a distance of just 350 m [60]. Another factor found in the literature for the use of bicycles was that it requires not only the design of infrastructure, but also a change of habits in the behavior of people. Thus, the trips registered in males are mainly for work reasons in comparison with females, whose trips are related to home and family support [61]. On the other hand, a study conducted in the city of Buenos Aires (Argentina) defined that 64% of users of public transport by bicycle were men, who perceived and valued aspects such as speed, economic savings, comfort, health, reliability, entertainment, and security of the service [62]. Finally, in the capital of Chile, it has been observed that 73.4% of bicycle users are males and 23.6% are females, where there is also a residential segregation due to a monocentric model, with public coverage for the use of bicycles that only reaches 14 of the 45 districts of the capital [63]. Therefore, although the distance determines the use of bicycles, there are other factors that must be taken into account for the promotion of this means of transport.

## 5. Conclusions

The non-active commuting mode is the most frequent form of mobility between high school and university, observing that active trips decreased when the distance from the home to the study center increased. It can also be observed that the AC modes (e.g. walking and cycling) decreased from the high school to the university, giving space to more passive modes of commuting, such as the metro/train and the bus. According to this, policies and strategies of public and private intervention are required, allowing to maintain or increase the modes of AC in the university stage, because this can become an opportunity to increase their physical activity, decrease the sedentarism, improve the quality of life in these young people, and favor an active life in adulthood.

## Figures and Tables

**Table 1:** General characteristics of participants (average and standard deviation/frequency) and differences between male and female students.

Characteristics	All (*n* = 1288)	Males (*n* = 614)	Females (*n* = 674)
	$\bar{X}$ ± SD	$\bar{X}$ ± SD	$\bar{X}$ ± SD
Age (years)	22.7 ± 5.8	25.31 ± 7.4	20.40 ± 2.1
***Socioeconomic Aspects***	***n* (%)**	***n* (%)**	***n* (%)**
Low	388 (30.1)	191 (31.1)	197 (29.2)
Medium	677 (52.6)	329 (53.6)	348 (51.6)
High	223 (17.3)	94 (15.3)	129 (19.1)
***University* (%)**			
UBB	87 (6.8)	36 (5.9)	51 (7.6)
UTFSM	475 (36.9)	318 (51.8)	157 (23.3)
PUCV	219 (17.0)	95 (15.5)	124 (18.4)
UDLA	507 (39.4)	165 (26.9)	342 (50.7)
***Area of studies* (%)**			
Administration	180 (14.0)	98 (16.0)	82 (12.2)
Basic Sciences	32 (2.5)	16 (2.6)	16 (2.4)
Sciences of health	288 (22.4)	89 (14.5)	199 (29.5)
Social sciences	59 (4.6)	23 (3.7)	36 (5.3)
Science of education	201 (15.6)	23 (3.7)	178 (26.4)
Engineering	472 (36.6)	332 (54.1)	140 (20.8)
Pedagogy in physical education	56 (4.3)	33 (5.4)	23 (3.4)

UBB: Universidad del Bío Bío; UTFSM: Universidad Técnica Federico Santa María; PUCV: Pontificia Universidad Católica de Valparaíso; UDLA: Universidad De Las Américas.

**Table 2:** Commuting mode that students use from their home to school and university.

Caption	Total	Males			Females		
		High School	University	*p*-Value ♂	High School	University	*p*-Value ♀
		*n* (%)	*n* (%)		*n* (%)	*n* (%)	
**Active Commuting**	466 (36.2)	244 (39.8)	209 (34.0)	**0.039**	222 (32.9)	125 (18.5)	**<0.001**
Walking	444 (34.5)	224 (36.5)	199 (32.4)	0.145	220 (32.6)	118 (17.5)	**<0.001**
Bicycle	22 (1.7)	20 (3.3)	10 (1.6)	0.052	2 (0.3)	7 (1.0)	0.180
**Non-active Commuting**	822 (63.8)	370 (60.3)	405 (65.8)	**0.039**	452 (67.1)	549 (81.4)	**<0.001**
Car	294 (22.8)	147 (23.9)	74 (12.1)	**<0.001**	147 (21.8)	101 (15.0)	**<0.001**
Motorcycle	3 (0.2)	3 (0.5)	6 (1.0)	0.453	0 (0.0)	1 (0.1)	**-**
Public bus	484 (37.6)	195 (31.8)	261 (42.5)	**<0.001**	289 (42.9)	378 (56.1)	**<0.001**
Metro/train	41 (3.2)	25 (4.1)	64 (10.4)	**<0.001**	16 (2.4)	69 (10.2)	**<0.001**

Statistical difference with *p* < 0.05; McNemar test.

**Table 3:** Commuting mode that students use from high school and university to home.

Caption	Total	Males			Females		
		High School	University	*p*-Value ♂	High School	University	*p*-Value ♀
		*n* (%)	*n* (%)		*n* (%)	*n* (%)	
**Active Commuting**	531 (41.2)	271 (44.1)	207 (33.7)	**<0.001**	260 (38.6)	119 (17.6)	**<0.001**
Walking	508 (39.4)	252 (41.0)	192 (31.3)	**<0.001**	256 (38.0)	114 (16.9)	**<0.001**
Bicycle	23 (1.8)	19 (3.1)	15 (2.4)	0.557	4 (0.6)	5 (0.7)	0.999
**Non-active Commuting**	757 (58.8)	343 (55.9)	407 (62.2)	**<0.001**	414 (61.5)	555 (82.6)	**<0.001**
Car	133 (10.3)	60 (9.8)	72 (11.7)	0.294	73 (10.8)	115 (17.1)	**0.001**
Motorcycle	5 (0.4)	4 (0.7)	5 (0.8)	1.000	1 (0.1)	1 (0.1)	1.000
Public bus	580 (45.0)	256 (41.7)	271 (44.1)	0.399	324 (48.1)	378 (56.1)	**0.002**
Metro/train	39 (3.0)	23 (3.7)	59 (9.6)	**<0.001**	16 (2.4)	61 (9.3)	**<0.001**

Statistical difference with *p* < 0.05; McNemar test.

**Table 4:** Probability of being active or non-active, according to distance from high school and university to home and vice versa.

	**To High School**					**To University**			
	***n***	**OR**	**95% CI**	***p*-Value**		***n***	**OR**	**95% CI**	***p*-Value**
***1.51–4 km (Q2)***					***2.1–6 km (Q2)***				
Active	156	1	Reference		Active	28	1	Reference	
Non-active	197	5.48	3.87–7.77	**<0.001**	Non-active	257	31.20	19.72–49.72	**<0.001**
***4.1–10 km (Q3)***					***6.1–15 km (Q3)***				
Active	40	1	Reference		Active	12	1	Reference	
Non-active	328	35.62	23.16–54.78	**<0.001**	Non-active	307	86.98	45.54–162.57	**<0.001**
>***10 km (Q4)***					>***15 km (Q4)***				
Active	4	1	Reference		Active	5	1	Reference	
Non-active	236	205.04	81.02–518.90	**<0.001**	Non-active	305	207.40	82.97–518.43	**<0.001**
	**From High School**					**From University**			
	*n*	**OR**	**95% CI**	***p*-Value**		*n*	**OR**	**95% CI**	***p*-Value**
***1.51–4 km (Q2)***					***2.1–6 km (Q2)***				
Active	179	1	Reference		Active	33	1	Reference	
Non-active	174	6.95	4.70–10.27	**<0.001**	Non-active	252	22.74	14.77–35.02	**<0.001**
***4.1–10 km (Q3)***					***6.1–15 km (Q3)***				
Active	59	1	Reference		Active	7	1	Reference	
Non-active	309	37.44	24.30–57.70	**<0.001**	Non-active	312	132.76	60.57–290.97	**<0.001**
>***10 km (Q4)***					>***15 km (Q4)***				
Active	7	1	Reference		Active	6	1	Reference	
Non-active	234	239.01	105.12–543.43	**<0.001**	Non-active	304	150.92	65.08–349.97	**<0.001**

OR = odds ratio; CI = confidence interval. The reference category for distance from school is from 0 to 1.5 km and the distance from the university is from 0 to 2 km. References adjusted by gender and socioeconomic status.
